# Telomere Abnormalities in the Pathobiology of Idiopathic Pulmonary Fibrosis

**DOI:** 10.3390/jcm8081232

**Published:** 2019-08-16

**Authors:** Hasancan Bilgili, Adam J. Białas, Paweł Górski, Wojciech J. Piotrowski

**Affiliations:** Department of Pneumology and Allergy, Medical University of Lodz, 90-154 Lodz, Poland

**Keywords:** telomere, telomerase, usual interstitial pneumonia, idiopathic pulmonary fibrosis, lung fibrosis

## Abstract

Idiopathic pulmonary fibrosis (IPF) occurs primarily in older adults and the incidence is clearly associated with aging. This disease seems to be associated with several hallmarks of aging, including telomere attrition and cellular senescence. Increasing evidence suggests that abnormalities involving telomeres and their proteome play a significant role in the pathobiology of IPF. The aim of this study is to summarize present knowledge in the field, as well as to discuss its possible clinical implications. Numerous mutations in genes associated with telomere functioning were studied in the context of IPF, mainly for Telomerase Reverse Transcriptase (TERT) and Telomerase RNA Component (TERC). Such mutations may lead to telomere shortening, which seems to increase the risk of IPF, negatively influence disease progression, and contribute to worse prognosis after lung transplantation. Some evidence indicates the possibility for the use of telomerase activators as potential therapeutic agents in pulmonary fibrosis. To sum up, increasing evidence suggests the role of telomere abnormalities in the pathobiology of IPF, natural history and prognosis of the disease. There are also possibilities for telomerase targeting in the potential development of new treatment agents. However, all these aspects require further research.

## 1. Introduction

Idiopathic pulmonary fibrosis (IPF) is a specific form of chronic fibrosing interstitial pneumonia of unknown etiology. The prevalence of this rare disease is estimated at 2–29 cases per 100,000 in the general population [1,2,3,4,5]. The natural history of the disease is characterized by a progressive decline of pulmonary function, as well as overall health and well-being, with a median survival time of 2–3 years [6,7,8,9].

Another interesting clinical issue is the familial occurrence of IPF, termed familial interstitial pneumonia (FIP). From 2 to 20% of patients with IPF have a positive family history. Inheritance appears to be autosomal dominant; however, with variable penetrance [10,11,12]. Steele et al. [13] reported that older age (68.3 vs. 53.1), male sex, and having ever smoked cigarettes were associated with the development of FIP. In addition, telomere abnormalities seem to contribute to the development of FIP.

IPF is limited to the lungs and is defined by the histopathologic and/or radiologic pattern of usual interstitial pneumonia (UIP). UIP’s histopathological image is described as patchy dense fibrosis that causes remodeling of the lung architecture, often resulting in honeycombing, and alternates with areas of less affected parenchyma. Honeycombing can be also seen in high-resolution computed tomography and is obligatory for definite radiological diagnosis of UIP [6].

However, UIP is not a pattern that occurs only in IPF and can be observed in other diseases, e.g., connective tissue diseases and drug-induced interstitial lung disease. Therefore, the diagnosis of IPF is rather by exclusion and we have to exclude other known causes of UIP pattern.

The fibrotic zones in UIP contain dense acellular collagen and scattered fibroblastic foci. Honeycomb is the end stage of fibrosis and is composed of cystic fibrotic airspaces frequently lined by bronchiolar epithelium and filled with mucin.

The other cells engaged in fibrosis are smooth muscle cells, which in this lesion are commonly hyperplastic [14,15].

IPF occurs primarily in older adults and the incidence is clearly associated with aging [16], which is also considered as one of the risk factors of the disease. Moreover, an increasing body of evidence suggests that premature ageing, primarily affecting the epithelial cells, would be one of the key issues in the pathobiology of the disease [17,18,19,20].

A telomere is a region of tandem repeats of short DNA sequences at the ends of chromosomes, which are important for their stability and allow the complete replication of the ends [21,22]. Telomere length homeostasis is essential for proper cellular function [22,23]. Telomeres are also crucial for chromosomal rearrangements or fusion prevention [24]. The telomere proteome consists of more than 200 proteins that are associated with different aspects of telomere functioning, including their protection, elongation, or telomeric DNA synthesis [21,25,26]. Abnormalities involving telomeres and their proteome seem to play a significant role in the pathobiology of IPF. The aim of this study is to summarize present knowledge in the field, as well as to discuss its possible clinical implications.

All names of genes and proteins were unified and presented following Gene Nomenclature Committee Guidelines [27].

## 2. Contribution of Telomere Abnormalities to the Etiology of IPF

IPF seems to be associated with several hallmarks of aging, including genomic instability, epigenetic alterations, loss of proteostasis, deregulated nutrient sensing, mitochondrial dysfunction, stem cell exhaustion, altered intercellular communication, telomere attrition, and cellular senescence [28,29].

Incomplete DNA replication causes the loss of small amounts of sequences which form telomeres at each S phase of the cell cycle; however, the enzyme telomerase compensates for this loss by the de novo addition of TTAGGG repeats [30]. Telomerase consists of telomerase reverse transcriptase (TERT), regulatory subunits and telomerase RNA component (TERC) [31]. Both these subunits were linked with lung fibrosis pathogenesis. Animal studies did not provide the evidence for spontaneous fibrosis in *Tert*^−/−^ or *Terc*^−/−^ mice [32,33]; however, there is evidence that suggests the role of such abnormalities in increasing susceptibility to lung fibrosis induced by bleomycin or lipopolysaccharide (LPS) [34,35]. There is also evidence of the role of *TERT* and *TERC* genes in the human population. Notably, heterozygous mutations in *TERT/TERC* were reported in ∼15% of patients with familial interstitial lung disease, as well as, but rarely in sporadic IPF (<3%) [36,37,38]. Additionally, Cronkhite et al. linked sporadic IPF with *TERT* or *TERC* no coding mutation, with 23% of the cases in this study showing said mutation. Such patients presented telomere lengths less than the 10th percentile when compared with control subjects. Even after controlling for age, sex, and ethnicity, suffering from pulmonary fibrosis was significantly associated with restriction of telomerase fragments. Overall, 25% of sporadic cases of pulmonary fibrosis had telomere lengths less than the 10th percentile [39]. It is also important to highlight that *TERC* mutations are associated with the shortest telomere lengths [40,41,42].

Sui et al. [43] reported that in addition to activating DNA damage pathways, shortened telomeres seem to contribute to fibrosis when exposed to an injurious stimulus, by impairing tissue repair. Chromosomal shortening resulting from telomere attrition activates checkpoint inhibitor p53. Activated p53, in turn, reduces mitochondrial biogenesis, increases reactive oxygen species production and activates cellular senescent pathways.

An increasing body of evidence supports the epithelial-mesenchymal hypothesis of IPF, which suggests that this disease is the result of successive injuries to the alveolar-capillary membrane with ineffective reconstitution of normal epithelium. The core of this hypothesis is that type II alveolar epithelial cells fail to repair the damaged epithelium as a result of ineffective proliferation, migration, and/or differentiation, and this leads to interstitial scarring [44]. This hypothesis can also be linked with the role of telomeres in IPF pathobiology. Namely, Snetselaar et al., based on the heterogenous character of fibrosis distribution in IPF, which allows for a comparison of telomere length between fibrotic and non-fibrotic tissue in a single surgical biopsy, found that it is in alveolar type 2 cells that telomere shortening is predominantly observed and associates with fibrotic lesions [45].

Short telomeres are not specific for IPF only. This pathology was described also in other interstitial lung diseases. However, it seems that telomeres in IPF are shorter than in other diseases from this group [41].

We should also highlight that the prevalence of interstitial lung diseases in carriers of telomere-related gene mutations increases with age [40].

Table 1 presents selected mutations associated with telomere abnormalities in idiopathic pulmonary fibrosis.

### 2.1. Smoking, Pulmonary Fibrosis and Telomere Shortening

Smoking is considered one of the risk factors of IPF [60]. There is also a well-documented interplay between smoke exposure and telomere shortening [61,62], which can partially be justified by the fact that smoking exposure contributes to oxidative damage [63]. Tsakiri et al. reported that the mean age of death of the smokers with a mutation in *TERT* or *TERC* was 10 years earlier than that of the nonsmokers [38]. Additionally, Morlá et al. showed that smoking causes telomere shortening in a dose-dependent manner [61].

Diaz de Leon et al. identified 134 individuals with heterozygous *TERT* mutations from 21 unrelated families. The authors concluded that there appears to be a significant association between smoking and/or fibrogenic exposures with pulmonary fibrosis in *TERT* mutation carriers who are ≥40 years of age [40].

According to Dressen et al., among self-reported ever-smokers with IPF and controls, both age and smoking status were associated with telomere length. Among all patients, in a model of telomere length by age, IPF diagnosis, and smoking status, IPF status was associated with telomere length, whereas a weaker association was observed between telomere length and smoking status [57].

### 2.2. Oxidative Stress and Mitochondria—Other Players in the Game

Telomere length is a function of its length at birth and its rate of attrition. The latter is a result of history of replication, in addition to reflecting various factors such as chronic inflammation and cumulative oxidative stress, acting on progenitor cells [64]. Increasing evidence demonstrates a pathogenetic role for oxidative/nitrosative stress in IPF. Increased oxidative stress may promote disease progression mainly in those who are current smokers [65]. The aberrant production of reactive oxygen species (ROS) and reactive nitrogen species (RNS) induces cellular damage. In IPF, both ROS and RNS amplify the transforming growth factor beta (TGF-β)—Mediated pathway through oxidation of redox-sensitive proteins. TGF-β induces mitochondrial oxidant radical formation in lung fibroblast by enhancing nicotinamide adenine dinucleotide phosphate (NADP^+^) oxidase, inhibiting sirtuin 3 expression and inactivating nuclear erythroid 2-related factor (Nrf-2) [66].

One of the endogenous sources of ROS is mitochondrial respiration. Mitochondrial electron transport chain even under normal conditions, may ‘leak’ 1–2% of all electrons as ROS. When mitochondria are damaged, ROS levels increase and begin to damage mtDNA. The damaged mtDNA continues to dysfunction the electron transport chain, which results in the enhanced generation of ROS, forming a kind of vicious circle [63]. Dysfunctional mitochondria were also linked with IPF pathobiology [19].

Another issue is the coexistence of insufficient antioxidant defense, which result in the inability to keep ROS below a toxic threshold. In IPF, significant reductions, e.g., in heme oxygenase (HO)-1 in broncho-alveolar lavage (BAL) alveolar macrophages, as well as downregulation of extracellular superoxide dismutase and pathways such as Nrf-2 in fibroblastic foci were reported [67,68].

Additionally, a significant part of oxidation processes occurs in the sulfhydryl group in proteins, resulting in protein misfolding. The accumulation of misfolded proteins in the endoplasmic reticulum is enhanced under conditions of oxidative stress and results in endoplasmic reticulum stress, which, together, leads to the malfunction of cellular homeostasis [69].

On the other hand, excessive and unsolved endoplasmic reticulum stress and mitochondrial dysfunction enhances an apoptotic response of the alveolar epithelial cells, which, due to the abnormal shortening of telomeres, have a deficient regenerative capacity. These processes, together with other aging-related changes, seem to be critical for the development of IPF [19].

### 2.3. Does Sex Make a Difference

Epidemiological data show that males suffer from IPF more frequently than women [70]. Additionally, females with IPF demonstrate better survival [71]. Moreover, this factor is an element of Gender-Age-Physiology (GAP) system, which seems to be a useful tool to predict mortality in patients with IPF [8,72].

Although there are no direct studies on human subjects in this field, prevalence and annual incidence of IPF increasing with age [16] intriguingly seems to correspond to age-related decline in testosterone level. There is no data supporting the above-mentioned hypothesis; however, considerable evidence suggests that sex hormones directly regulate telomerase [73,74]. Moreover, Townsley et al. reported that androgens were efficacious in the treatment of inherited bone marrow failure associated with telomere dysfunction, producing clinically meaningful hematologic improvement. Authors also observed that the increase in telomere length seen in patients treated with hormones was consistent with hormone-mediated up-regulation of TERT and of telomerase enzymatic activity [75].

The above mentioned lacks in knowledge, indicating a strong need for further research in this field.

### 2.4. Familial Interstitial Pneumonia

Telomere abnormalities seem to contribute to the development of FIP. Cronkhite et al. observed the significantly higher proportion of probands with familial pulmonary fibrosis (24%) in which no coding mutation in *TERT* or *TERC* was found, had telomere lengths less than the 10th percentile when compared with control subjects. Overall, 37% of familial cases of pulmonary fibrosis in this study had telomere lengths less than the 10th percentile [39]. The association between *TERT* and/or *TERC* mutations was also reported by Tsakiri et al. Heterozygous carriers of *TERT/TERC* mutations had shorter telomeres than age-matched family members with wild type [38].

On the other hand, Armanios et al. [37] reported that six of 73 probands (8%) had heterozygous mutations in human *TERT* or *TERC*. Mutant telomerase resulted in short telomeres. Moreover, asymptomatic subjects with mutant telomerase also had short telomeres, suggesting that they may be at risk for the disease. Authors also concluded that telomere length may serve as a surrogate marker for the identification of patients at greatest risk for carrying mutant telomerase genes, whereas longer telomeres appeared to predict the absence of a telomerase mutation. However, these observations require verification in larger samples.

## 3. Telomere Abnormalities and the Natural History of IPF

Telomere abnormalities seem to be associated not only with the risk of pulmonary fibrosis, but also contribute to the natural history of the disease.

In the context of pulmonary fibrosis, telomere shortening has been described as a negative prognostic factor [76]. Diaz de Leon et al. observed that pulmonary fibrosis associated with *TERT* mutations was progressive, with reduced life expectancy [40]. Similarly, Dressen et al. assessed samples from 1510 patients with sporadic IPF from three international phase 3 clinical trials (INSPIRE, CAPACITY, ASCEND) and 1874 non-IPF controls. Three percent of 1046 patients with an rs35705950 risk allele in the intergenic region between TOLLIP and MUC5B had a rare protein-altering variant in *TERT* compared with 7% of 464 non-risk allele carriers (OR 0.4; 95% CI: 0.24–0.66). Subsequent analyses identified enrichment of rare protein-altering variants in Poly(A)-Specific Ribonuclease (*PARN*), Regulator of Telomere Elongation Helicase 1 (*RTEL1*), and rare variations in *TERC* in patients with IPF compared with controls. The proportion of patients with at least one rare variant in *TERT, PARN, TERC*, or *RTEL1* was higher in patients with IPF than in controls. Patients with IPF who had a variant in any of the four identified telomerase component genes had telomeres that were shorter than patients without a variant in any of the four genes and had an earlier mean age of disease onset than patients without one or more variants. Moreover, in the placebo arms of clinical trials, shorter telomeres were significantly associated with faster disease progression [57].

## 4. Telomere Length and Lung Transplantation in IPF

Although an increasing body of evidence shows undisputable benefits from using antifibrotic drugs [77,78,79,80,81,82,83], lung transplantation is still the only therapy that has been clearly shown to prolong survival of patients with IPF [84,85]. However, International Society for Heart and Lung Transplantation (ISHLT) highlight that lung transplantation should be considered for adults with chronic, end-stage lung disease who also present enough satisfactory high likelihood of post-transplant survival [86]. While IPF undoubtedly fulfils most of these criteria, the latter part of the statement raises some concern. The perspective provided by the broad context of the above presented evidence shows the depreciative effect on survival of IPF patients. That is why, in our opinion, this issue deserves a separate chapter and broader discussion.

Silhan et al. [87] described a series of lung transplants in the U.S.A., Australia and Sweden, that combined to eight subjects with the median age at transplantation of 52 years. Enrolled patients carried a mutation in telomerase or had the diagnosis of a telomere syndrome, defined as previously, and confirmed by abnormally short age-adjusted telomere length. Five subjects in this group carried mutations in *TERT*, two subjects had mutations in *TERC*, and one fulfilled clinical telomere syndrome criteria, a positive history of thrombocytopenia, a family history of pulmonary fibrosis, but no detectable mutation in *TERT, TERC or DKC1*. Hematological complications were the most prevalent morbidity in this group and required significant adjustment of the immunosuppressive regimen in all patients. Other complications reported by authors were the high rate of acute renal failure and acute tubular necrosis, gastrointestinal bleeding that required red blood cell transfusion support, infectious complications in several subjects, and one patient presented defective healing at the anastomotic site. In addition, in some patients there was impaired functional recovery, with three individuals developing critical illness polyneuropathy and myopathy. Despite the high frequency of complications, seven of the eight patients were alive with a median follow-up of 1.9 years, and one subject died 10 months post-transplant after a prolonged hospitalization.

Another important issue discussed by Silhan et al. [87] is the risk for rare medication-related toxicities among patients with telomere defects. This problem deserves close attention, as patients after lung transplantation are exposed to a number of cytotoxic medications as part of the immunosuppression or anti-microbial prophylaxis regimen.

Borie et al. [88] reported nine patients with *TERT* or *TERC* mutations with a median age of 52 years at the time of lung transplantation. The authors observed the following post-transplant complications: myelodysplasia and/or bone marrow failure, anemia and neutropenia. The median survival after lung transplantation was 214 days.

On the other hand, Newton et al. analyzed 82 patients and reported that having telomere length <10th percentile was independently associated with worse survival as well as a shorter time to onset of chronic lung allograft dysfunction with more frequent grade three primary graft dysfunction. On the contrary of previous reports, there was no difference in the incidence of acute cellular rejection, cytopenias, infection, or renal dysfunction among short telomere patients.

To sum up the above presented evidence, we can conclude that telomere shortening seems to be associated with increased medical complications. This may be justified by the overall pathobiological landscape of this particular disease group, notably, their multi-systemic manifestation. Multiple organs and tissues are affected, including different epithelia and bone marrow. This characteristic manifestation includes IPF patients and may well explain the origin of the complications presented after lung transplantation. Although, there is a piece of evidence about worse survival and shorter time to onset of chronic lung allograft dysfunction, which would justify screening IPF patients for telomere length prior to qualification, routine implementation of such examination requires further research.

## 5. Telomere Length and Antifibrotic Treatment

There are only data on pirfenidone treatment in the context of telomere length. Dressen et al. observed a significant interaction between telomere length and treatment on lung function decline. Shorter telomeres at the study baseline timepoint predicted more rapid forced vital capacity (FVC) decline in patients with IPF than did longer telomeres, and treatment with pirfenidone had benefit regardless of telomere length status [57].

Additionally, a recent study showed that neither pirfenidone nor nintedanib modulated the expression of senescence markers [89].

## 6. Targeting Telomerase in Therapy of IPF

As of now, there is no such treatment registered for use in the human population; however, Povedano et al. presented an interesting perspective in this field [35]. The authors reported the potential therapeutic use of adeno-associated vectors (AAV) to transiently activate telomerase in adult tissues. They showed therapeutic effects in a mouse model of pulmonary fibrosis owing to a low-dose bleomycin insult and short telomeres. AAV9 preferentially targets regenerative alveolar type II cells (ATII). The use of AAV9-*Tert* therapy resulted in improved lung function and lower inflammation and fibrosis at 1–3 weeks after viral treatment, and improvement or disappearance of the fibrosis at eight weeks after treatment. AAV9-Tert treatment led to longer telomeres and increased proliferation of ATII cells, as well as lower DNA damage, apoptosis, and senescence [35].

Elsewhere, Le Saux et al. used a GRN510 molecule (derived from cycloastragenol) to activate telomerase. The authors observed that GRN510 can reduce fibrosis following bleomycin administration in animal models, suggesting that GRN510 may prove effective as a potential therapeutic agent for IPF [90].

## 7. Conclusions

To sum up, increasing evidence suggests the role of telomere abnormalities in the pathobiology of IPF, natural history, and prognosis of the disease. There are also possibilities for telomerase targeting in the potential development of new treatment agents. However, all these aspects require further research.

## Figures and Tables

**Table 1:** Selected mutations linked to Idiopathic pulmonary fibrosis (IPF) and/or familial interstitial pneumonia (FIP) with references.

Gene	Function	OMIM Number	Mutation	Reference Sequence	Amino Acid Substitution	Comment	Ref.
*TERT*	Enzyme in telomerase complex	187270	97C>T	coding DNA	Pro33Ser		[38]
			164T>A	coding DNA	Leu55Gln		[37]
			277+1G>A	coding DNA			[37]
			(334_336)delC	coding DNA	Pro112ProfsX16		[37]
			430G>A	coding DNA	Val144Met		[38,46]
				coding DNA	Val170Leu	uncertain description	[47,48]
			1234C>T	coding DNA	His412Tyr		[49]
			1456C>T	coding DNA	Arg486Cys		[38]
				coding DNA	Arg622His	uncertain description	[50]
			1885G>C	coding DNA	Gly629Arg		[51]
			1989G>C	coding DNA	Ser663Arg		[51]
				coding DNA	Val664Leu	uncertain description	[48]
			2069G>C	coding DNA	Trp690Ser		[51]
			2081C>T	coding DNA	Val694Glu		[51]
			2110C>T	coding DNA	Pro704Ser		[51]
			2225C>T	coding DNA	Arg742His		[51]
			2240delT	coding DNA	Val747AlafsX20		[38]
			2329G>A	coding DNA	Val777Leu		[50,52]
			2383-2A>G	coding DNA			[49]
			2583-2A>C	coding DNA	Leu862_Leu884del		[37]
			2593C>T	coding DNA	Arg865Cys		[38]
			2594G>A	coding DNA	Arg865His		[38]
				coding DNA	Arg865Ala	uncertain description	[46]
				coding DNA	Thr874Arg	uncertain description	[46]
			2620A>G	coding DNA	Thr874Ala		[51]
			2648T>G	coding DNA	Phe883Cys		[53]
			2812C>T	coding DNA	Arg938Trp		[51]
				coding DNA	Phe1032Ile	uncertain description	[50]
			3323C>T	coding DNA	Pro1108Leu		[49]
			3329C>T	coding DNA	Thr1110Met		[37]
			3346_3522del177	coding DNA	Glu1116fsX		[38]
*PARN*	mRNA stability	604212		coding DNA	Phe8LeufsX	uncertain description	[48]
			246-2A>G	coding DNA			[46,54]
			529C>T	coding DNA	Gln177X		[54,55]
			563_564insT	coding DNA	Ile188IlefsX7		[54]
			14676110C>A	coding DNA; genomic	Glu374X	uncertain description	[51]
			1262A>G	coding DNA	Lys421Arg		[54]
			14647946G>C	coding DNA; genomic	Leu461Val	uncertain description	[51]
			14540858CCT>C	coding DNA; genomic	Glu585AspfsX5	uncertain description	[51]
*RTEL1*	DNA helicase	608833	62320919A>AC	coding DNA; genomic	Arg675ThrfsX15	uncertain description	[51]
			623321112G>A	coding DNA; genomic	Gly703Arg	uncertain description	[51]
			2214-2A>C	coding DNA			[51]
			62321477T>C	coding DNA; genomic	Trp751Arg	uncertain description	[51]
			62321483C>T	coding DNA; genomic	Arg753Cys	uncertain description	[51]
			2992C>T	coding DNA	Arg998X		[51]
			62324600C>T	coding DNA; genomic	Arg1010X	uncertain description	[51]
			602delG	coding DNA	Gly201GlufsX15		[54]
			1451C>T	coding DNA	Pro484Leu		[54]
			1940C>T	coding DNA	Pro647Leu		[54]
			2005C>T	coding DNA	Gln693X		[54]
			2695T>C	coding DNA	Phe899Leu		[49]
			2824G>A	coding DNA	Asp942Asn		[49]
			2875C>T	coding DNA	His959Tyr		[49]
*NAF1*	catalyzes the addition of repetitive DNA sequences to telomeres	617868	956_957delAA	coding DNA	Lys319Argfs*21		[56]
			984insA	coding DNA	Ser329Ilefs*12	PF combined with emphysema	[56]
*DKC1*	telomerase stabilization and maintenance	300126	1213A>G	coding DNA	Thr405Ala		[57]
					**Region**		
*TERC*	Template in telomerase complex	602322	37a>g	RNA	P1b		[38]
			98g>a	RNA	P2b		[37]
			108c>u	RNA	P3		[50]
			325g>u	RNA	P5		[47]
			375_377delgga	RNA	Box H	PF combined with emphysema	[58]

Modified on the basis of the Telomerase Database [59]. Abbreviations of gene names: *DKC1*—Dyskerin; *NAF1*—Nuclear assembly factor 1 ribonucleoprotein; *RTEL1*—Regulator of telomere length (helicase) 1; *TERC*—Telomerase RNA component; *TERT*—Telomerase reverse transcriptase.
